# Cardiovascular Risk Reduction With Icosapent Ethyl: A Systematic Literature Review

**DOI:** 10.7759/cureus.10942

**Published:** 2020-10-14

**Authors:** Mala Thakur, Pritpal S Sangha, Areesha Satti, Pooja N Shah

**Affiliations:** 1 Internal Medicine, Xavier University School of Medicine, Oranjestad, ABW; 2 Medicine, Medical University of Lublin, Lublin, POL; 3 Internal Medicine, Saint James School of Medicine, The Valley, AIA

**Keywords:** diabetes mellitus, cardiovascular, icosapent ethyl, awareness of cardiovascular disease, heart disease, statins, cholesterol, triglycerides, lipoproteins

## Abstract

Cardiovascular disease is the leading cause of death in the United States of America due to elevated triglyceride levels. High triglyceride levels lead to higher risks of ischemic events. There are multiple pieces of research and analyses on statin therapy and its ability to reduce the prevalence of heart complications. Heart ailments can reduce through the use of icosapent ethyl in the form of statin therapy. This literature review will explain the reduction of cardiovascular risks with icosapent ethyl. Though some genetic conditions can cause some of these ailments, the rest of the predisposing conditions revolve around cholesterol, lipoproteins, and triglycerides.

## Introduction and background

Introduction

Cardiovascular diseases have several predisposing conditions, which include smoking, lack of exercise, obesity, age, etc. These conditions can lead to a situation that gives rise to heart issues or conditions that foster an environment that can encourage these risks. Several predisposing factors can lead to cardiovascular disease. These factors are smoking, poor diets, obesity, low or high low-density lipoprotein (LDL) or high-density lipoprotein (HDL) cholesterol figures, obesity, as well as a known history of cardiac ailments in a family [1]. Each of these factors touches on the issue of fat, lipids, or, in other words, triglycerides.

The problem of cholesterol, specifically low-density lipoproteins, is an essential underlying condition propelling most heart-risk illnesses. Every factor, prevalent disease, and even predisposing condition is related to the human body's cholesterol level. From poor diets to obesity, almost all situations have a trigger that correlates to cholesterol levels. This issue goes as far as smoking. Nicotine and other substances in cigarette smoke raise LDL levels while concurrently promoting the increase of triglycerides within blood [1]. Therefore, beyond making the heart and blood vessels narrow, cigarette smoke causes the narrative to always point towards cardiovascular risk whenever the aspect of cholesterol runs unchecked. This detail is in sharp contrast to instances where research shows that HDL lipoproteins have a positive influence on the prevention of arteriosclerosis [2]. Since arteriosclerosis is the most prominent indicator of the chance of heart illness, it, therefore, points out that unchecked lipoproteins and triglycerides are critical in eradicating cardiac risk in society.

The icosapent ethyl compound within the drug known as Vascepa shows several positive impacts as well as controls bad lipoproteins and triglycerides. Icosapent ethyl has multiple effects that encourage a reduction in the prevalence of heart issues. This drug works in the manner of a plasma-binding protein in the blood. The medicine in the body works with a combination of triglycerides, cholesterol, and phospholipids. This medication works as a statin drug in that it works to reduce cholesterol levels in the body [3]. By lowering cholesterol and the chemicals that induce its formation, these medicines help reduce the risk of cardiac illnesses. Icosapent ethyl, however, stands out from the many statin drugs in terms of overall efficiency in reducing the risk of heart-related sicknesses.

Background of the study

There are multiple pieces of research and analyses on statin therapy and its ability to reduce the prevalence of heart complications. One such research piece took place in Asia. The Asian population happens to be the community most prone to getting premature heart complications, together with diabetes. The role of statins as the best or most appropriate form of cardiac risk reduction remains inconclusive. A 2013 review on the mortality rate among the general population within the community as a result of cardiac-related issues supports the value of statin medication towards treating and gradually eradicating heart diseases [4]. This mortality rate in question also appears in the form of a lower figure of the significant cardiovascular severe events and the medical procedures associated with artery revascularization in hospitals and health centers.

Statin therapy also shows its influence on cardiac issues, such as myocardial infractions, death, stroke, and, to no small extent, the expenses incurred due to stents, angioplasties, and bypass surgery. In comparison, Asian Indian people have a very high risk of coronary artery illness. They also have a similarly high risk of diabetes. This fact is gradually attributed to their lifestyle, along with a well-known insulin resistance predisposition. The environment that they live in is also a contributing factor that points to the predisposing factors stated above. These issues revolve around the aspect of their diet, which is high in sugar, refined, and processed foods. The people also have a smoke and pollution issue, which, when combined with the lack of inactivity, both affect the aspect of cholesterol and triglycerides [5]. However, this risk factor is closely associated with the spread of non-communicable diseases around South Asia. 

Statin therapy is applied in clinical trials against cardiovascular diseases. These drugs work by inhibiting 3-hydroxy 3-methylglutaryl coenzyme A reductase. This enzyme plays a crucial role in the synthesis of cholesterol in the body. This medication also affects the magnitude of low-density lipoproteins associated with cholesterol. Atherosclerosis is an illness that is closely related to the presence of lipoproteins and triglycerides in the body. This form of treatment helps in the overall eradication of cardiac ailments within the general public. This ideal, however, is spearheaded by the availability of icosapent ethyl as the drug of choice. 

Similar research on cardiovascular diseases appears in a study to assess the effectiveness of oral semaglutide. This compound is a peptide receptor agonist drug given to patients with type two diabetes and has a high risk of heart-related complications [6]. The research carried out revealed that for the 3000 participants, the cardiovascular risk significantly reduced through the use of the drug. PIONEER (Peptide Innovation for Early Diabetes Treatment), through its treatment of diabetes, also proved to reduce illnesses and cardiac-related issues, steadily reducing heart-related complications among nearly 86% of the participants for almost 14 years [6].

Further study on cardiovascular illnesses leads to the use of empagliflozin. Researchers carried out a test dubbed the EMP-AREG OUTCOME (BI 10773 (Empagliflozin) Cardiovascular Outcome Event Trial in Type 2 Diabetes Mellitus Patients) trial to test the effectiveness of this drug to treat heart failure in the more significant part of society [7]. This health measure is another drug that falls under the same sodium-glucose transporter inhibitor class that proves useful when combined with diet and exercise. In these steps, the findings of this study further show the correlation between diabetes and heart complications. This drug plays a similar function: its ability to regulate hyperglycemia and blood pressure in victims who have diabetes, which also treats cardiovascular diseases. 

Problem statement

Heart ailments can be reduced through the use of icosapent ethyl in the form of statin therapy. Cardiac illnesses can not only be managed but also treated through the use of this drug. Diabetes, lifestyle choices, and cholesterol influxes also add to the element of cardiac risks in society. By reducing the causative agents that lead to the initiation of cardiovascular illnesses, the rate of heart-related mortalities can reduce from the high 30% of mortality it currently stands at. The use of statin therapy in the form of icosapent ethyl will significantly reduce the influence of low-density lipoproteins and triglycerides within the blood system.

Aims

This paper aims to bring to light the correlation between the issues of cholesterol and cardiovascular ailments. Though some genetic conditions can cause some of these ailments, the rest of the predisposing conditions revolve around cholesterol, lipoproteins, and triglycerides. Several studies allude to the fact that cholesterol levels majorly affect an individual's ability to suffer from cardiovascular issues. The predisposing conditions all draw their origin from the lifestyle and environmental triggers surrounding cholesterol production. The lipids and facts manufactured by individuals who have a poor diet, inadequate nutrition, and even diabetes tend to cause heart diseases. The studies that point to this topic show that when the levels of these fats, enzymes, and lipoproteins are regulated, the state of cardiac ailments reduces simply by changing the lifestyle and choices one makes. The only way to control these substances is by using drugs such as Vascepa, whose active ingredients icosapent ethyl help inhibit and regulate the cholesterol enzymes. This study also aims to show that the use of icosapent ethyl has more merits than demerits. 

Specific objectives

1. Show the relevance of triglycerides and low-density lipoproteins in the prevalence of cardiovascular ailments.

2. Show the change and influence of the lifestyle choices we make and how they affect heart disease.

3. Show the positive impact of the use of icosapent ethyl. 

Significance of the study

This study aims to shed light on the particulars affecting cardiovascular ailments. Rather than simply state them, the research seeks to find the causative agents that correlate with each individual's predisposing conditions. The study should enlighten the reader on the link between diabetes and cardiac illnesses, mainly why type two diabetes is the most common of all contributing risk factors. This research also helps to show the value and specific attributes that the icosapent ethyl possesses that make it stand out among most of the statin medications offered in the market. The health and side effects are also a cause for concern in this paper, as the relevant resources only present the definite form of icosapent ethyl medication. Therefore, one should even understand the corresponding side effects that come from this renowned drug that is set to help treat and manage cardiac conditions.

Methodology

The use of icosapent ethyl is steadily coming up as a solution to the eradication of cardiovascular illnesses within society. This proposal for treatment emanates from the fact that this drug has proven to lower triglyceride levels and, therefore, reduce cardiac incidences. A study on participants suffering from cardiac ailments, diabetes, and a triglyceride level of 135-499 mg per deciliter supports this inference [8]. These participants also had to have a low-density lipoprotein level of 41-100 mg for every deciliter. The 8,179 patients each received a dose of two grams of icosapent ethyl two times a day. The choice of action was to curb the related heart diseases such as stroke, myocardial infractions, and cardiovascular fatalities. 

This sample population comprises a total of 19,212 patients that came for the eligibility screening tests. For this study, however, only 8,179 participants were selected. Researchers arrived at this number after 152 of the total patients failed to complete the final visits and another 578 refused to give consent. The rest of the failed participants did not appear on the list of participants due to a myriad of other reasons. Of the total participants selected, 70% had a cardiac illness while 29% had a cardiac disease but with added risk factors such as those of diabetes mellitus, among many more. The participants had a mean age of approximately 64 years [8]. The study aimed to test the results of cardiovascular fatalities, myocardial infarctions, unstable angina, and many more.

The participants all had two forms of statin therapy. These treatments have a broad structure to best fit the two groups of participants, namely, the icosapent ethyl group and the placebo group. The icosapent ethyl group had a daily dose of 4 grams icosapent ethyl while the placebo group had a placebo that resembles icosapent ethyl. This placebo contained mineral oil to better look like icosapent ethyl. As researchers had to adhere to the integrity of research, a randomization technique selected the participants. All these protocols and procedures ran as per design in a trial dubbed the Reduction of Cardiovascular Events using icosapent ethyl, REDUCE-IT, which was primarily an intervention trial. 

Databases

**Figure 1:**
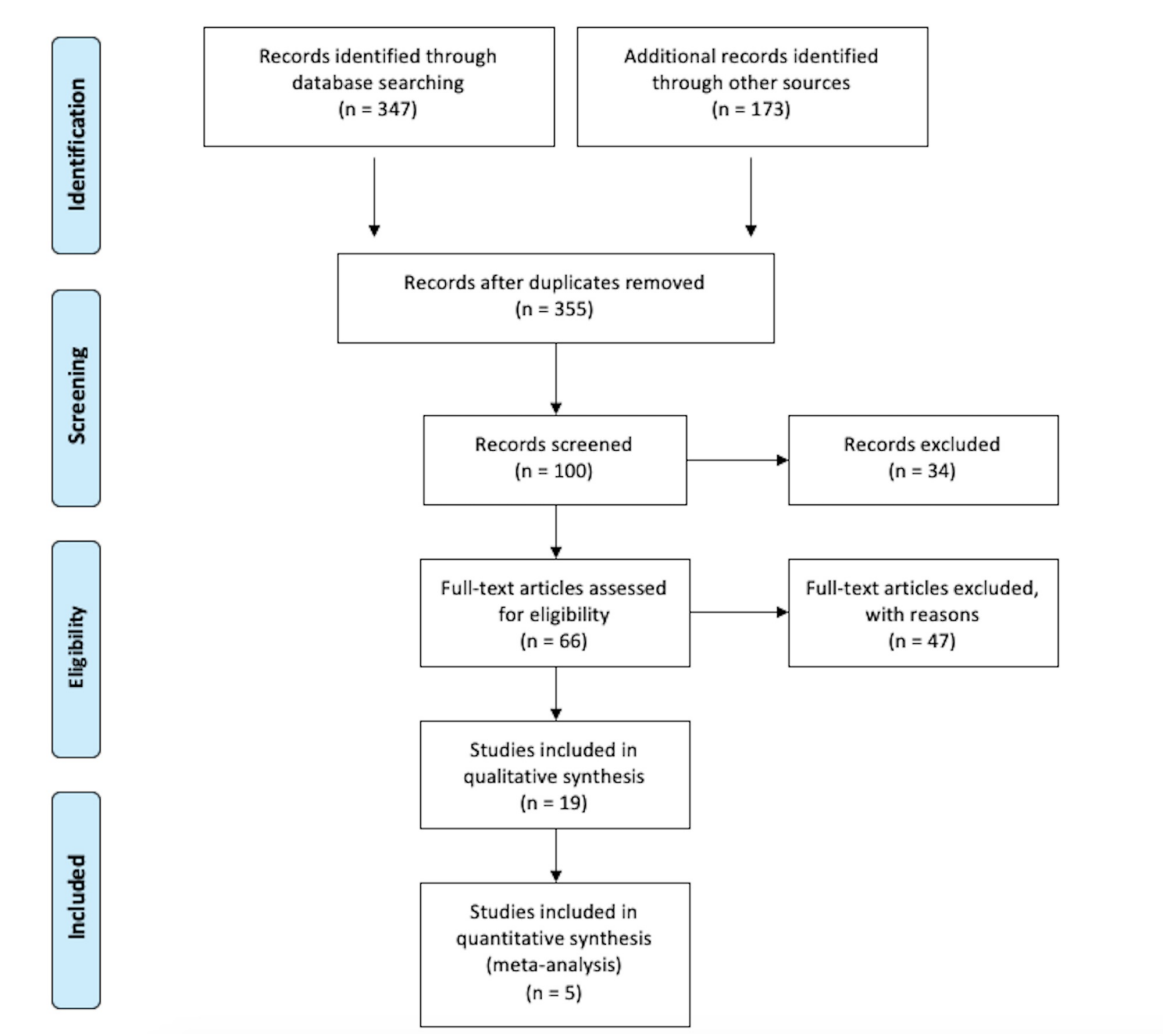
PRISMA 2009 flow diagram: cardiovascular risk reduction with icosapent ethyl: a literature review PRISMA: Preferred Reporting Items for Systematic Reviews and Meta-Analyses Source: [9]

To obtain the information needed, a thorough search of four databases was conducted as illustrated in Figure 1. The Research Gate, National Center for Biotechnology Information, PubMed, and Google Scholar databases were used to identify peer-reviewed articles relevant to the research topic. A total number of 347 records were identified as suitable based on the search words used. Other sources, such as school newsletters and the World Health Organization, were also used to identify peer-reviewed articles. This search yielded 173 records from the search words used. All duplicated articles were removed.

Eligibility

Records were screened to ensure that information was pertinent to the topic at hand. Records were screened for information in the English language, publication between 2008-2020, and peer review. Articles with small sample sizes were not included. Articles that focused on multiple comorbidities were not included to limit the research on diabetes and cardiovascular issues.

## Review

Literature review

The plague of cardiovascular illnesses has led to millions of fatalities. According to the World Health Organization (WHO) figures, heart ailments kill about 17.9 million lives every year [1]. This illness is the number one reason for death around the world. These cardiovascular diseases include rheumatic, arrhythmia, and other related cardiac disorders. Almost 80% of cardiovascular deaths are due to strokes and cardiac arrest in people below 70 years. These individuals tend to have high blood pressure and are overweight or obese. Though these health factors undergo vigorous testing in most of the primary care facilities, the top-most risk-taking measures are appropriate. Access to medicine and health technologies within hospitals helps those in need to be treated and manage their conditions [3].

The prevalence of diabetes is an added risk factor for the presence of cardiovascular illnesses. Type two diabetes has almost a two to a four-fold higher rate of mortality. This data is, however, before the avenues for reassessment after the introduction of cardiovascular disease reduction approaches. This study uses approximately 963,000 participants who receive treatment from the United States Veterans Health Care System. This research was from 2002 to 2014, followed by a medical visitation and subsequent appointments for eight years. Those in charge of the study followed the aspects of type 2 diabetes and hemoglobin in the blood system [10]. When put under covariate incidence rates and hazard regressions, these factors resulted in assessing cardiovascular disease death among the subjects tested.

Almost 34% of the group participants tested positive for diabetes mellitus. These participants had a cardiac illness mortality figure of 3.5 deaths out of 1000 people as compared to the regular deaths from standard age factors. When other factors, such as age, race, and ethnic surroundings, factored in, the risk of cardiac-related fatalities decreased while that of diabetic-related illnesses increased. These figures, however, show that overall, given the period of the study and subsequent eight years for a follow-up, participants who had diabetes had a 7% higher chance of dying from cardiac-related complications [11]. The hemoglobin tests carried out also show the lowest mortality regardless of the cardiovascular history that the patients may have had. This study proved inconclusive when the test parameter was hemoglobin-based, but the overall presence of type 2 diabetes appears to increase heat-related deaths significantly. Hemoglobin may not have a direct relation to the death rates of the participants, yet it still plays a role in predicting the presence of cardiovascular ailments.

A study on the prevalence of atherothrombosis among diabetes patients over four years also proves a direct correlation to cardiovascular illnesses. In this study, it is apparent that issues such as myocardial infractions and non-fatal strokes were common among patients with diabetes mellitus. This research proves that individuals who have diabetes also tend to have cardiovascular illnesses [12].

There also remains another similarity between the presence of diabetes and cardiovascular illnesses. This instance appears in the preemptive cardiac disease of atherosclerosis. Atherosclerosis is the most common condition associated with cardiac ailments. The narrowing of the arteries is an issue that affects blood pressure, often causing strokes, cardiac arrest, among many more problems. Atherosclerosis, however, is an issue related to the occurrence of diabetes as well. Atherosclerosis helps correlate the two ailments supporting the ideology that cardiovascular disease and diabetes have the same source. This source can either be genetically or environmentally linked [13]. The environment one is born into, as well as the nutrition one exposes themselves to, is a leading contributor to both diabetes and cardiovascular illness.

In treating diabetes, researchers have also found that medication is given to patients also serves a purpose in the reduction of cardiac-related illnesses. One such instance appears in the use of sodium-glucose inhibitors. These inhibitors are a regular occurrence in diabetes medicine. Their availability links directly to their role in regulating hyperglycemia, body weight, and blood pressure [14]. Coincidentally, these symptoms are predisposing factors to most of the known heart-related complications.

A diabetes-related condition, such as insulin resistance, emanates from a lack of tolerance for glucose and primarily dyslipidemia. Dyslipidemia is a condition where the body grapples with high-density lipoproteins, varying cholesterol levels, and high or low triglyceride levels [15]. Although these factors remain highly controversial in the medical fraternity, their similarity to cardiac-related illnesses is undeniable. The appearance of these elements and factors in both diabetes mellitus and cardiovascular diseases may be epidemiologically arguable. Still, the simple fact that they appear links the two illnesses closely together. Further studies show a distinct relationship between blood lipoproteins and lipids as a cause of hereditary heart failure [14]. This research shows loci in the genetic structure of the cells that correspond to the lipids, triglycerides, cholesterol, and many more type two diabetes-related factors that contribute to cardiovascular disease.

Patients that are suffering from high triglyceride levels are more prone to cardiac illnesses. This inference is a fact proven through several studies and research. A 1996 study showed that even though there lies a steady high-density lipoprotein level, which is a decisive factor against cardiac ailments, high triglyceride levels can still cause cardiovascular diseases. Scholars have since built on this study and come up with facts to show the value of plasma triglyceride levels in predicting heart disease. This fact purports the idea that plasma triglycerides can best predict the risk of a cardiac-related attack or illness.

Current factors in western society have embraced the use of hydroxy methylglutaryl coenzyme A reductase inhibitors to curb cardiac complications in the community. The purpose of this method has shown results, but on a large scale, the fatality reduction was not as expected. The numbers anticipated were to be much higher than they currently are. This gap in results has led to the continued search for a more efficient solution that can provide the desired reduction in cardiovascular deaths. These subdued results come about because the inhibitors could not wholly eradicate the negative lipoproteins within the blood plasma. As the rated number of obese individuals worldwide increases, high-density lipoprotein cholesterols increase, but so does the number of small low-density lipoproteins. Therefore, the use of plasma triglycerides predicts the rise in heart-related illnesses [16]. Triglycerides, therefore, are a factor promoting the spread of coronary heart-related disease. Non-fasting forms of triglycerides offer an addition to the fact of cardiac illness treatment by quickening assessment and prediction when analyzed. 

Icosapent ethyl also has an effect on the blood and plasma within the body. The purity level of this eicosapentaenoic acid ensures that it infiltrates the blood plasma and cells to better cope with the essential role of triglyceride and lipoprotein management. Researchers use an exploratory analysis of the red blood cells and blood plasma to gauge the effectiveness of this drug and constituent element. This test focuses on the red blood cell membrane and the concentration of fatty acids before and after exposure to icosapent ethyl [17]. When the triglyceride levels within a body have lower figures, some things change. The first is a change in the blood plasma's fatty acids while the second is the change of the substances within the red blood cell lining. The blood's fatty acid concentration showed no shift in omega-six but a difference in the red blood cell membrane lining, which shows a direct change in the levels of triglyceride levels.

The size and number of lipoprotein particles can also reduce when one uses icosapent ethyl. A study aimed to show the efficiency and effective performance of icosapent ethyl when pitted against other drug compounds, such as atorvastatin and simvastatin, among others, used with a combination of ezetimibe. These medications were given for 12 weeks after the participant's system was clear of all previous statin drugs using a four- to nine-week period of cleansing as the body cleaned out the drugs from its system. After 12 weeks, the participants were given a dose of 4 grams a day by the doctors in charge. The lipoprotein particles were analyzed using nuclear magnetic resonance spectroscopy.

The sample size picked comprised individuals that were 65 years and below and were overweight young men who had type two diabetes as well. The results showed that the selected participants stayed within their baseline values with no significant increase or decrease in particle size. Triglyceride values and high-density lipoproteins did reduce as a result of the statin therapy that the participants were going through. This figure represents the control group. A similar program was also running, where the participants used icosapent ethyl for twelve weeks. Each participant was given the Food and Drug Administration (FDA)-approved 4 gram a day dose [18]. In this group, however, there was a sharp reduction in the size and concentration of low-density particles. While all the other values for the lipoprotein size reduced, the low-density particles, however, increased. However, these values do not take away from the fact that atherogenic lipoprotein concentration significantly reduces after using the required icosapent ethyl dose for the given period.

Results

These study participants had to undergo steady observation for approximately five years, with only 17% of the patients under the icosapent ethyl dosage regiment having a heart-related disease or complication. The participants that were the control experiment had a dosage of the placebo, and almost 22% of them developed a cardiac complication within the five years of research. These results show that the patients that had icosapent ethyl showed less risk of cardiac events. This inference shows that those who had a standard placebo during this time showed a 5% increase in cardiac instances. The influence of icosapent ethyl in the reduction of triglyceride levels was apparent on the tests that followed as the study progressed. The triglyceride levels reduced by approximately 18%, which translates to a loss of 39 milligrams for every deciliter. This figure is in sharp contrast to the placebo group, where there was an actual increase of 4.5 milligrams for every deciliter [8]. There was an increase in the negative form of lipoprotein, but the icosapent ethyl group still has a lower number than their placebo counterparts. The increase in low-density lipoproteins was around 3% in the icosapent group while the placebo group increased by 10.2%. These results show that compared to the standard placebo in statin therapy, icosapent ethyl works well to lower triglyceride levels while reducing the involuntary production of low-density lipoproteins in the body.

In terms of the health and side effects of this drug, there were no significant disparities between the two groups. Individuals in the icosapent ethyl group suffered from pneumonia, but the same number of people tested positive for the illness as well in the placebo group. Atrial fibrillation and peripheral edema were other issues resulting from these test groups, but the instances were higher in the icosapent ethyl group than in their counterparts. Other matters were anemia, diarrhea, gastrointestinal complications, flutters, and severe bleeding in some cases within the icosapent ethyl group, even though no fatalities resulted from this bleeding.

Discussion

Icosapent ethyl is an example of eicosapentaenoic acid. Eicosapentaenoic acid is a form of omega-3 acid. This fatty acid is present in fish species that have a lot of fat, such as salmon. In cases of manufacture, this compound is also present in fish oil supplements alongside docosahexaenoic acid. Omega 3 acids are commonly known to help fight heart disease throughout the world [19]. When added to one's diet, there is a significant reduction in coronary heart ailments, triglyceride levels above average, and even high blood pressure. These compounds are responsible for reducing the occurrence of blood clots within the body and improving arterial health by reducing arterial plaque within the arterial channels, which tends to clog up the vessels restricting blood flow. Most people suffering from heart complications receive advice from their general practitioner to have fat fish at least twice a week to introduce these omega-three acids into their diets.

Icosapent ethyl is an example of this form of organic omega-3 acid. As it comes from this source, it possesses the same influence and ability to work on the regulation of triglyceride levels along with the reduction of negative lipoprotein increase. This drug's impact is so practical and thoroughly proven that the United States Food and Drug Administration approved it to reduce cardiovascular ailments. While other drugs can also lower triglyceride levels, the single gap filled by icosapent ethyl is that it can significantly reduce triglyceride levels without always increasing the levels of harmful cholesterol. This attribute is a feat that most drugs cannot claim to do. Other drugs within the statin range of therapy and treatment tend to lower triglyceride levels. They inadvertently raise the low-density lipoproteins, which is counterproductive, as these lipoproteins also contribute to the prevalence of cardiovascular illnesses. 

This exceptional trait in the icosapent ethyl compound, coupled with its already strong eicosapentaenoic acid origin, makes this the most appropriate drug to reduce and manage cardiac ailments. The drug's ability to target the triglyceride levels shows, while leaving the other compounds just as they are, that it focuses on the direct trigger for heart disease without elevating other predisposing factors. This targeted approach to cardiovascular disease eradication promotes general health and wellness of the patients that take it. The welfare is further held in place because most of the side effects from the drug are mild with some gastric while others are bleeding. Still, the other heart-related conditions all emanate from the already established heart conditions. These features combine to make icosapent ethyl the drug of choice both against type two diabetes as well as cardiovascular treatments and eradication.

Limitations

This study focuses on the use and merits of the active ingredient icosapent ethyl. This focus narrows down the number of statin drugs in question otherwise in the market and serves in a similar capacity. Therefore, this frame of thought can be one-sided and lacks a comparison drug to show the true extent of the impact. This research also focuses on type two diabetes over type one. This choice to only select the model two comes from the fact that type two diabetes results from lifestyle patterns rather than the latter form of diabetes that is primarily genetic. Early type two diabetes is also reversible, unlike type one that is permanent from birth. The use of statin therapy through tested and reviewed results-wise shows positive feedback in treating this form of diabetes. The use of only one type of this disease constricts the scope to just a section of diabetic patients rather than all diabetic patients. This conclusion, therefore, waters down the universal appeal for the drug on all thing's diabetes.

## Conclusions

Cardiovascular illnesses and diabetes have a lot in common. They are both triggered by the fact that varying triglyceride levels exist along with low- and high-density variations of lipoproteins within the blood. These compounds are the exact elements that icosapent ethyl targets in functioning as a form of statin therapy. The superiority of icosapent ethyl comes from the fact that though this drug significantly lowers the available triglycerides, the lipoprotein levels are left intact or minutely raised. Other medications may lower triglyceride levels, but they involuntarily raise the low-density lipoprotein levels, which counteracts all the progress made in the process of the cardiac disease since low-density lipoproteins encourage the prevalence of cardiac ailments. Icosapent ethyl is the best-suited medication for fighting against cardiovascular risk and conditions in the world.
